# Subcutaneous Sarcoidosis during Pegylated Interferon Alfa and Ribavirin Treatment for Chronic Hepatitis C

**DOI:** 10.1155/2010/230417

**Published:** 2010-04-29

**Authors:** R. Rodríguez-Lojo, M. Almagro, J. M. Barja, F. Piñeyro, L. Pérez-Varela, J. Del Pozo, M. T. Yebra-Pimentel, E. Fonseca

**Affiliations:** ^1^Department of Dermatology, Complexo Hospitalario Universitario A Coruña, 15006 A Coruña, Spain; ^2^Department of Pathology, Complexo Hospitalario Universitario A Coruña, 15006 A Coruña, Spain

## Abstract

Interferon is used to treat hepatitis C virus infection and its cutaneous side effects are well known. Recently, interferon-induced sarcoidosis has been reported. We report a new case of sarcoidosis during pegylated interferon alfa and ribavirin treatment with an unusual presentation in a woman with previous episodes of erythema nodusum and nodular vasculitis related to HCV.

## 1. Introduction

Sarcoidosis is a systemic granulomatous disorder characterized by the presence of noncaseating granulomas. Its precise pathogenesis remains unclear but several cytokines, like interferon, may play a key role in the formation of granulomas.

Interferon is used to treat hepatitis C virus (HCV) infection due to its antiviral and immunomodulating properties and its cutaneous side effects are well known (Table 1) [1], being localized inflammatory skin reactions the most common.

Recent reports have noticed the development of sarcoidosis in patients receiving the combination of pegylated interferon alfa and ribavirin for the treatment of hepatitis C. In some cases there were only skin lesions but extracutaneous involvement was detected in other patients. We report a case of subcutaneous sarcoidosis developed during interferon alfa and ribavirin combined therapy for HCV infection.

## 2. Case Report

**Figure 1:**
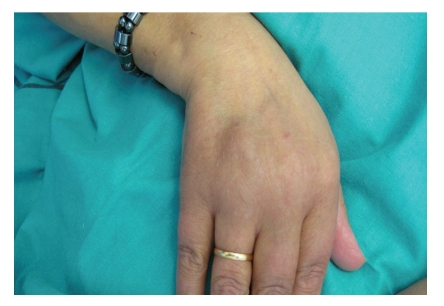
Subcutaneous nodule on the hand.

A 60-year-old woman was infected by HCV (genotype 1, stage II/IV) after a blood transfusion in 1979. Treatment with subcutaneous pegylated interferon alfa (180 mcg weekly) and oral ribavirin (800 mg daily) was started in April 2007. Tolerance was poor by fatigue, weight loss (7 kg), and depressive syndrome. After six months of treatment she suffered a cutaneous eruption of painful, mobile, small, and subcutaneous nodules on her arms and legs (Figure 1). She had history of several episodes of subcutaneous lesions biopsied as erythema nodosum and recurrent nodular vasculitis.

A skin biopsy revealed multiple noncaseating granulomas in the septal and lobulillar adipose tissue consistent with sarcoidosis. Special stains for bacterial, fungal, and mycobacterial organisms were negative. Laboratory studies demonstrated a moderate lymphopenia (2,57 × 10^9^/L), anemia (hemoglobine 10.2 g/dL), and elevated angiotensin-converting enzyme (62 UI/L). The remaining studies (chest X-ray, thoracic and abdominal computed tomographic, pulmonar function testing, calcium serum levels, as well as tumoral and inflammatory markers) showed no data of systemic sarcoidosis.

Treatment was discontinued and the skin lesions cleared after 2 months. Hepatitis remained stable with undetectable viral load and normal liver enzymes.

## 3. Comment

Chronic hepatitis C affects at least 170 million people worldwide [2] and the cutaneous lesions related to HCV are very diverse. Several types of panniculitis, such as nodular vasculitis and erythema nodosum, have been reported [3]. 

Treatment regimens used for chronic hepatitis include interferon alfa (IFN-*α*) monotherapy, INF-*α* plus ribavirin, and most recently peginterferon alfa, which has replaced classic IFN-*α* as the first-line drug in combination with ribavirin, specially in more resistant viral genotypes 1, 4, 5, and 6 [4, 5]. Pegylated interferon alfa, synthesized by adding a polyethylene glycol molecule to the standard interferon structure, has antiviral, antiproliferative, and immunomodulating properties [3, 5]. 

The use of IFN-*α* is associated with several side effects (fatigue, fever, myalgia,…) that occur in 40%–55% of patients, and cutaneous events (pruritus, rash,…) reported in up to 20% of patients [3]. It may induce in addition some autoimmune diseases (tiroiditis, lupus erythematosus,…) [4, 6–8]. Sarcoidosis is another recognized adverse effect of IFN-alfa.

Sarcoidosis is a systemic disease characterized by the presence of noncaseating granulomas. Although its exact etiology remains unclear, it is thought to represent an exaggerated immune response to antigenic stimuli (infections, malignancies, environmental factors,…) [4, 6, 9, 10]. Cell immunity is involved and cytokines like interferons lead a clonal proliferation of T lymphocytes and differentiation of macrophage cells into epithelioid cells; both mechanisms play an important role in the genesis of granuloma [4, 6]. 

In sarcoidosis there appears to be a predominance of a helper T cells Type 1 (Th1) immune response and Th2 lymphocytes are relatively inactivated in the granulomas. IFN-*α* stimulates the differentiation of Th1-type lymphocytes and reduction of the activation of Th2 lymphocytes, favoring the formation of granulomas in susceptible patients [4, 6, 7]. Pegylated interferon alfa was found to be superior to classic IFN-*α* for enhancement of Th1-immune response and it increases the risk of developing sarcoidosis when compared with standard IFN-*α* [11].

Ribavirin is a nucleoside analog of guanosine that enhances the Th1 response and inhibits Th2 production [3, 5, 6]. It explains that combination therapy with IFN-*α* and ribavirin is more effective in treating hepatitis C virus and it also may further predispose patients to sarcoidosis.

We think that upregulation of the Th1 immune response by pegylated interferon alfa and ribavirin in the presence of an antigenic trigger may play a key role for the induction of sarcoidosis in susceptible individuals. HCV may act as an antigenic trigger. Since 1987 when the first case of pulmonar sarcoidosis following interferon therapy was published, there have been published more than 20 observations of sarcoidosis related to interferon, in most of them combined with ribavirin.

The time to the onset of interferon-induced sarcoidosis ranges from 15 days to 30 months after the starting of treatment. Men and women are equally affected [4, 6]. Skin manifestations appear in more than 50% of cases, and patches are the most common form of presentation, but other dermatological signs (subcutaneous nodules, scar infiltration,…) have been reported. Nondermatological symptoms were also observed, being pulmonar involvement the most frequent [4–8]. However, these symptoms are nonspecific and can resemble the usual side effects of IFN-*α*; then it is possible that interferon-induced sarcoidosis is underestimated. This underlines the importance of dermatological examination that can provide helpful diagnosis.

Usually interferon-induced sarcoidosis follows a benign course. In some cases cutaneous lesions were resolved without treatment in a few months and it was possible to continue hepatitis treatment with careful follow-up and close monitoring of systemic problems. However, there are cases where discontinuation of interferon therapy is necessary and even treatment with systemic steroids [4, 10].

In summary, it is known that HCV infection may be associated with many dermatologic diseases. Our patient had recurrent episodes of erythema nodosum and nodular vasculitis associated with HCV. Development of sarcoidosis may be related to an antigenic trigger such as HCV in a susceptible patient with an enhanced Th1 response from pegylated interferon and ribavirin.

 We report a case of interferon-induced sarcoidosis with an unusual presentation as subcutaneous nodules, without extracutaneous involvement, and it resolved completely after interferon was withdrawn.

## Figures and Tables

**Table 1:** Cutaneous side effects of interferon.

Most common	Pruritus, xerosis, eczema, and localized inflammation at injection site
Relatively common	Psoriasis, lichen planus, and vitiligo

Isolated	Eosinophilic pustular folliculitis, erythematosus lupus, Meyerson's naevi, facial erythema, hypopigmented atrophic plaques, hyperpigmentation, alopecia, Sweet's syndrome, calcified nodules, sclerodermatous graft versus host disease, cutaneous necrosis, fixed drug eruption, rheumatoid artritis, panniculitis, pemphigus foliaceus, Raynaud's phenomenon, vasculitis, and urticaria
